# CIRSE Standards of Practice for the Interventional Radiology Management of Acute and Chronic Arterial Mesenteric Ischaemia

**DOI:** 10.1007/s00270-025-04080-0

**Published:** 2025-07-10

**Authors:** Romaric Loffroy, Antonio Basile, Edit Dósa, Geert Maleux, Bora Peynircioglu, Olivier Chevallier

**Affiliations:** 1 Department of Vascular and Interventional Radiology, François-Mitterrand University Hospital, Dijon, France; 2 Dipartimento di Scienze Mediche, Chirurgiche e Tecnologie avanzate, Unità Operativa di Radiologia 1 Policlinico Universitario G.Rodolico, Catania, Italy; 3 Heart and Vascular Center, Semmelweis University, Budapest, Hungary; 4 Department of Radiology, University Hospitals KU Leuven, Leuven, Belgium; 5 Hacettepe University, Ankara, Turkey

**Keywords:** Mesenteric ischaemia, Percutaneous transluminal angioplasty, Stents, Superior mesenteric artery, Surgical revascularisation

## Abstract

**Purpose:**

Mesenteric ischaemia due to arterial occlusion, usually secondary to atherosclerosis or arterial thromboembolism, may be acute or chronic. Endovascular intervention is now the primary treatment based on its minimally invasive nature and lower in-hospital mortality. Open surgery is indicated in the presence of bowel necrosis, the diagnosis of which remains challenging. This document makes best-practice recommendations about the interventional radiology management of acute and chronic mesenteric ischaemia.

**Methods:**

The CIRSE Standards of Practice Committee established a writing group of six clinicians with internationally recognised expertise in the management of arterial mesenteric ischaemia. The writing group performed a pragmatic search on PubMed for relevant studies published in English in 2013–2024. The final recommendations were developed by consensus.

**Results:**

Endovascular interventions for arterial mesenteric ischaemia have very high technical success rates. Complication rates are lower than with open surgical revascularisation; however, patency times may be shorter. Patients must receive antiplatelet therapy and be monitored for recurrence.

## Introduction

The CIRSE Standards of Practice Committee established a writing group which was asked to produce up-to-date recommendations for the Interventional Radiology management of acute and chronic arterial mesenteric ischaemia. CIRSE Standards of Practice documents are neither clinical practice guidelines nor systematic reviews of the literature. They seek to recommend best practices rather than to impose a standard of care. Institutions should regularly review their internal development and improvement procedures in the light of new international guidance, local resources, and regular internal morbidity and mortality reviews.

A summary of key recommendations for the Interventional Radiology management of acute and chronic arterial mesenteric ischaemia is provided in Table 1.

**Table 1:** Summary of key recommendations for the interventional radiology management of acute and chronic arterial mesenteric ischaemia

**Acute mesenteric ischaemia**
**Imaging**
CTA without delay, including pre-contrast, arterial, and venous phases
• Diagnostic of arterial occlusion
• Signs of bowel necrosis
**Treatment recommendations**
Multidisciplinary approach
Close haemodynamic monitoring; Aggressive fluid resuscitation may be required
Intra-vascular volume expansion with crystalloids and blood products must be started immediately to improve visceral perfusion
Close monitoring of electrolyte and pH values and correction of any abnormalities
First-line endovascular revascularisation for patients with stable haemodynamics and no CTA evidence of transmural necrosis or peritonitis
Endovascular treatments
• Atherosclerosis: Stenting
• Embolism: Thrombus aspiration
Additional procedures may be required (catheter-directed thrombolysis, stenting)
Bowel resection if signs of bowel necrosis
**Post-treatment and follow-up care**
Dual antiplatelet therapy for at least 1 month, then lifelong antiplatelet monotherapy
Anticoagulant therapy: 6 months, or lifelong if required by another condition
Immediate post-operative parenteral anticoagulation after bowel resection in patients with AMI
Clinical monitoring
Follow-up imaging by Duplex US at 1, 6, and 12 months, then annually

**Chronic mesenteric ischaemia**
**Imaging**
Ultrasound and Duplex US: Does not obviate the need for CTA
CTA is essential: Stenosis assessment
**Treatment recommendations**
Symptomatic patients with multi-vessel disease and
• > 50% stenosis of the SMA
• > 70% stenosis of the CA
If single-vessel disease (CA or SMA), stenosis > 70% is required
CA angioplasty or stenting **should not be performed** in patients with active compression by the median arcuate ligament
Endovascular treatment: Stenting
• SMA recanalisation is the primary goal
• Covered preferable to bare-metal stents
**Post-treatment and follow-up care**
Dual antiplatelet therapy for 1 month, then lifelong antiplatelet monotherapy
Clinical monitoring
Follow-up imaging by Duplex US or CTA

*AMI* Acute mesenteric ischaemia; *SMA* Superior mesenteric artery; *CA* Coeliac artery

## Methods

The writing group, established by the CIRSE Standards of Practice Committee, consisted of six clinicians with internationally recognised expertise in the management of acute and chronic arterial mesenteric ischaemia. The writing group reviewed the literature on the topic by performing a pragmatic evidence search using PubMed. Relevant publications in the English language published between 2013 and 2024 were assessed. Relevant older sources were included where the data have not been updated and clear gaps were evident.

## Background

Mesenteric ischaemia, defined as insufficient blood supply to the bowel, is potentially life-threatening, impairs quality of life, and can generate high healthcare costs. Imaging studies are crucial not only to establish the diagnosis but also to identify the cause. Endovascular interventions play a substantial role as they are both effective and minimally invasive; however, the management strategy should be tailored to the cause, site involved and, most importantly, acute or chronic pattern of ischaemia. Venous and non-occlusive aetiologies are not considered in this SOP document.

### Acute Mesenteric Ischaemia

Acute mesenteric ischaemia (AMI) is defined as the sudden abruption of blood flow in a mesenteric artery causing ischaemia, cell damage, and necrosis of the corresponding bowel segment. If not treated rapidly, AMI can be fatal. This is a rare condition accounting for only 1/2000 acute hospital admissions per annum in Europe and the US [1]. Abdominal pain is the usual presentation and, given the lack of specificity of this symptom and low incidence of the disease, the diagnosis can be challenging. Failure to make a prompt diagnosis carries a risk of bowel necrosis and death [2].

The prevalence and aetiologies of the two principal mechanisms of AMI have changed in recent years, with acute arterial thrombosis due to atherosclerosis now more common than acute arterial thromboembolism. Emboli originating in the heart usually accumulate at the narrowest point of the arteries and are associated with concurrent emboli to other organs. The superior mesenteric artery (SMA) is the most frequently involved site due to its large calibre and acute take-off angle. Most emboli lodge within a 10-cm segment starting 3 cm distal to the SMA origin. In contrast, mesenteric thrombosis usually occurs at the origin of the artery, where atherosclerotic plaque narrows the lumen. Other causes of mesenteric artery occlusion include vasculitis, aortic dissection, mesenteric artery dissection, and mycotic aneurysms [3]; the specific management of these conditions will not be discussed.

The classic clinical presentation of AMI is sudden-onset, severe abdominal pain without proportional physical findings such as evidence of peritonitis (e.g., guarding or rebound tenderness). Ischaemia initially affects the mucosa, causing the pain. A high level of suspicion is required to establish the diagnosis at this stage. The eventual development of signs of peritonitis usually indicates irreversible ischaemia with bowel necrosis. Other symptoms at presentation may include nausea, vomiting, and diarrhoea with bloody stool. Septic shock may occur if the diagnosis is delayed [3, 4]. Acute-on-chronic mesenteric ischaemia is also common, notably in hospitalised patients.

The treatment of AMI was surgical until the development of endovascular techniques. Primary endovascular management in all patients with AMI remains controversial due to the lack of strong scientific evidence regarding outcomes and the high risk of death in AMI; however, a meta-analysis concluded that both mortality and bowel resection rates were lower with endovascular techniques than with open surgery [5] favouring an endo-first approach in the absence of bowel necrosis. There is no existing consensus regarding a diagnostic and therapeutic algorithm; thus, a multidisciplinary approach is optimal. Selection of a treatment strategy is based on the stage of ischaemic damage, notably the presence of bowel necrosis.

First-line endovascular revascularisation is currently indicated for patients with stable haemodynamics and no CT evidence of transmural necrosis or peritonitis. The main treatment goal is to restore blood flow to the ischaemic segment before transmural necrosis occurs. Ischaemia starts at the mucosa and progressively extends to the serosa, leading to transmural infarction. Irreversible intestinal damage typically occurs within 6 h in cases of complete vascular occlusion [6]. When signs of intestinal necrosis and peritonitis are present, irreversible bowel damage is highly likely and surgery is the first-line treatment [3, 7]. Consequently, these signs should be viewed as contra-indications to endovascular revascularisation alone. Nonetheless, debate continues regarding the optimal management at this stage, as revascularisation is still required and improves the chances of salvaging ischaemic but non-necrotic bowel segments. The main point of contention is the relative timing of revascularisation and bowel resection. The management at this stage varies across centres, reflecting the lack of a consensus.

### Chronic Mesenteric Ischaemia

Chronic mesenteric ischaemia (CMI) is defined as insufficient blood supply to the bowel responsible for ischaemic symptoms over at least 3 months. Stenosis or occlusion of two or more main mesenteric arteries, coeliac artery (CA), SMA, and inferior mesenteric artery (IMA) was previously thought to be required for symptoms to develop. There is now increasing recognition that involvement of a single artery may result in symptoms if collateral flow is limited. The very low prevalence of CMI reflects the abundance of collaterals in the gastrointestinal vascular system [8, 9]. Atherosclerosis of the mesenteric arteries has a reported prevalence of 6–29% but only rarely causes ischaemia [8, 10].

Atherosclerosis is the leading cause of CMI, which is most common in females and elderly individuals. Recognised risk factors include smoking, hypertension, diabetes, hypercholesterolaemia, high-fat diet, and low level of physical activity. Median arcuate ligament syndrome, vasculitis, and fibromuscular dysplasia are other causes of occlusive arterial CMI [11]; the specific management of which will not be addressed in this SOP.

The classic symptom triad consists of post-prandial pain, weight loss due to fear of eating, and diarrhoea. The pain is usually described as dull cramps that start shortly after eating and last 1–2 h [12]. Most patients eat smaller portions to avoid the pain. As the condition worsens, patients may avoid eating and experience substantial weight loss; however, since the development of computed tomography angiography (CTA), the diagnosis is usually achieved before major weight loss develops. Requiring the classic triad of symptoms to be present would therefore result in diagnostic and treatment delays [10]. The post-prandial timing of the pain is related to the increased blood supply needed during digestion in the CA, SMA, and IMA territories. As the disease progresses, the collateral flow becomes insufficient to supply the needs of the bowel at rest and the pain becomes continuous. Severe fatigue and diarrhoea are common at this stage. This condition is referred to as acute-on-chronic mesenteric ischaemia and carries a risk of acute bowel infarction [8, 13].

Abdominal pain and weight loss occur in many conditions including abdominal malignancies, chronic pancreatitis, duodenal ulcers, and coeliac disease. A multidisciplinary approach is therefore mandatory to optimise the diagnostic process. The compatibility of the history and imaging findings of significant stenosis with a diagnosis of CMI should be discussed and alternative aetiologies excluded. The number of occluded or stenotic mesenteric arteries is important when selecting the most appropriate treatment. Most current guidelines indicate that unexplained abdominal pain with significant stenosis in both the CA and the SMA warrant a diagnosis of CMI and suggest revascularisation; however, in single-vessel disease, a more comprehensive approach is needed and, in addition to imaging studies, functional tests, such as tonometry and visible light spectroscopy, should be considered [10, 13].

The definition of significant mesenteric artery stenosis is debated. Guidelines issued by the Society of Interventional Radiology and Society for Vascular Surgery define a significant stenosis as greater than 70% narrowing of the luminal diameter [13, 14]. European guidelines, however, consider the surface area and post-prandial flow rate [10]. As these are both greater in the SMA than in the other mesenteric arteries, SMA stenosis greater than 50% is considered haemodynamically relevant when multiple arteries are involved. Treatment is therefore indicated in symptomatic patients with multi-vessel disease and > 50% SMA stenosis or with > 70% stenosis of either the SMA or the CA [10]. A score for mesenteric artery stenosis severity reported in 2022 considered not only the number of affected vessels and the degree of narrowing or occlusion but also the length of the lesion and the presence of multiple stenoses in the involved arteries [15]. While this classification was described based on a normal anatomic pattern, the authors suggested that it could also be applied in the presence of anatomic variations, such as the absence of one of the visceral arteries. Of note, some patients with occlusion of both the CA and the SMA have few or no symptoms, due to compensation by IMA collaterals. IMA stenosis in this situation becomes an indication for endovascular treatment.

The goals of treatment include symptom relief, improved quality of life, weight restoration, and improved survival via prevention of bowel infarction. Traditional surgical revascularisation produces good outcomes including high long-term patency rates, with primary patency reported at 91–94% at 1 year and 80–81% at 5 years [16, 17]. Nonetheless, endovascular revascularisation is now the primary treatment method as the hospital stay is shorter and in-hospital mortality is lower. Limited evidence has not yet confirmed that long-term patency matches that of surgery and further studies, ideally randomised controlled trials, are needed.

*Relative contra-indications* to endovascular treatment are heavily calcified lesions and lesions caused by extrinsic compression. Surgery may also be considered in younger patients, as current data suggest longer patency times after mesenteric bypass [13, 18].

Median arcuate ligament syndrome (Dunbar syndrome), which results from active compression of the CA by the median arcuate ligament, is considered a contra-indication to endovascular repair [18]. However, CA stenting may be performed if the median arcuate ligament has been surgically released beforehand [18].

Open surgical mesenteric revascularisation is generally reserved for patients in whom endovascular treatment is not considered appropriate due to unfavourable mesenteric lesions, or for whom endovascular treatment has failed, or in cases of in-stent restenosis or occlusion [10, 18]. For patients who have undergone percutaneous stenting, endovascular reinterventions can be carried out until they are no longer possible and the only option is surgical revascularisation, in which case endovascular treatment represents a bridge to surgery. For some patients with a severely diseased SMA presenting with CMI, endovascular treatment of the CA or IMA may be considered as a bridge to open surgery [13]. This may also apply to some malnourished patients who may initially be considered unfit for open surgery. Attempts to improve nutritional status prior to revascularisation may have negative consequences, aggravating digestive ischaemia [10]. Revascularisation remains the priority, and thus, the endovascular approach also provides a bridge to surgery in this setting, yielding longer-term benefits.

## Patient Preparation

### Clinical Evaluation, Laboratory Tests, and Imaging

Tables 2 and 3 summarise the clinical findings, laboratory tests, and imaging techniques for AMI and CMI, respectively.

**Table 2:** Acute mesenteric ischaemia: Patient preparation, clinical evaluation, laboratory tests, and imaging

**Acute mesenteric ischaemia**	
***Patient profile*** [3, 19–22]	Elderly; Patients at high risk for • Embolic events: Cardiac or aortic disease (i.e., atrial fibrillation, valve disease, or left ventricular dysfunction) • Thrombotic events: History of CMI • Non-occlusive mesenteric ischaemia: Critically ill patients with hypotension and/or hypovolemia • Venous mesenteric ischaemia: Insidious onset, may be asymptomatic; Often complicates hypercoagulable states
***Clinical findings*** [19, 23]	Sudden severe abdominal pain; Pain intensity >> physical findings • Abdomen may remain soft and non-tender in the early phase Non-specific and inconsistent signs • Vomiting • Diarrhoea • Abdominal distension • Bloody stool Signs of peritonitis: Irreversible ischaemia, transmural bowel infarction/necrosis
***Laboratory tests*** [24–28]	Cannot establish the definitive diagnosis of AMI Common findings • Leucocytosis • Metabolic acidosis with lactate elevation Other biomarkers [23–25] • D-dimer elevation • Inflammatory markers elevation (IL-6, IL-8, C-reactive protein, and procalcitonin) • Intestinal fatty acid-binding protein elevation
***Imaging*** [3, 29–31]	Plain radiography • Very limited role in the early diagnosis of AMI • Intestinal pneumatosis in severe cases • Free intra-abdominal air if perforation is present Ultrasound and Duplex US • Not routinely performed when AMI is suspected • Lack sensitivity for visualising the distal mesenteric artery territory (emboli) Computed tomography angiography • Without delay when AMI is suspected • Pre-contrast phase: Intra-mural haemorrhage • Arterial and venous phases • Diagnostic of arterial occlusion • Early ischaemic changes in the bowel wall • At a later stage: signs of bowel necrosis • Absent bowel wall enhancement • Bowel wall thinning • Bowel wall haemorrhage • Mesenteric fat stranding • Pneumatosis intestinalis • Portal or mesenteric venous gas • Ascites • Free intra-abdominal air (bowel perforation) • Multiplanar reconstructions are helpful • Anatomy and origin of the mesenteric arteries assessment Dual-energy CT • 40-keV imaging • Increased contrast between normally enhanced segments and ischaemic non-enhancing segments • Iodine mapping • Decreased mucosal iodine uptake in ischaemic bowel • More susceptible to artefacts

*CMI* Chronic mesenteric ischaemia; *AMI* Acute mesenteric ischaemia

**Table 3:** Chronic mesenteric ischaemia: Patient preparation, clinical evaluation, laboratory tests, and imaging

**Chronic mesenteric ischaemia**	
***Patient profile*** [8–11]	Atherosclerotic patients • Most common in females and elderly • Risk factors • Smoking, hypertension, diabetes, hypercholesterolaemia, high-fat diet, and low level of physical activity Median arcuate ligament syndrome, vasculitis, and fibromuscular dysplasia
***Clinical findings*** [10, 12, 13, 32, 33]	Classic triad of symptoms (inconsistently present) • Post-prandial pain • Weight loss (fear of eating) • Diarrhoea Rule out differential diagnoses • Abdominal malignancies and chronic pancreatitis • Upper gastrointestinal endoscopy, colonoscopy, abdominal imaging studies
***Laboratory tests***	No laboratory tests exist for assisting in the diagnosis of CMI
***Imaging*** [10, 13, 34]	Plain radiography • Non-contributory in CMI Ultrasound and Duplex US • Often first-line imaging investigation to rule out other abdominal diseases • Useful to assess mesenteric artery occlusive disease • Does not obviate the need for CTA Computed tomography angiography • Essential • Assess the grade of stenosis • Diagnostic of CMI, in presence of multi-vessel disease and compatible clinical presentation • > 50% stenosis of the SMA • > 70% stenosis of the CA • If single-vessel disease (CA or SMA), stenosis > 70% is required

*CMI* Chronic mesenteric ischaemia; *SMA* Superior mesenteric artery; *CA* Coeliac artery

## Treatment

### Acute Mesenteric Ischaemia

The management of AMI requires a multidisciplinary approach involving gastroenterologists, vascular and abdominal surgeons, interventional radiologists, and intensive care physicians [35]. Once AMI is diagnosed, pre-operative critical care is mandatory [3]. Close haemodynamic monitoring is crucial due to the risk of haemodynamic compromise, volume loss, and third spacing. Aggressive fluid resuscitation may be required, potentially exceeding 10 L of IV fluids in the first 24 h of treatment [36], taking care to avoid volume overload [37]. Intra-vascular volume expansion with crystalloids and blood products must be started immediately to improve visceral perfusion [3, 38]. Vasopressors should be considered only as a last resort for refractory hypotension, due to their potential to induce intestinal vasospasm, and should be avoided whenever possible [32, 37]. The risk of severe metabolic acidosis and hyperkalaemia after bowel infarction and reperfusion requires close monitoring of electrolyte and pH values and correction of any abnormalities [39]. Nasogastric decompression should be initiated. Early, broad-spectrum antibiotic therapy is required due to the high risk of infection secondary to bacterial translocation [3, 40]. Intravenous full-dose anticoagulation, preferably with unfractionated heparin, should be initiated unless contra-indicated [3]. Although no specific recommendations exist regarding anticoagulation targets in acute arterial mesenteric ischaemia, an activated clotting time (ACT) between 250 and 300 s should be targeted by analogy with most peripheral intra-arterial endovascular procedures. Supportive care should include symptomatic treatments such as antiemetics for nausea and analgesics for pain relief.

In the acute setting, numerous revascularisation techniques are available, the choice of which depends chiefly on the cause of arterial occlusion: stenting is used for atherosclerosis, while thrombus aspiration (also known as aspiration embolectomy) or catheter-directed thrombolysis are employed for emboli. In certain cases, these techniques may be used in combination.

The SMA and/or CA can be reached via the femoral, brachial or, more rarely, the radial approach [41–43]. If there is an acute downward angle between the aorta and SMA/CA or if the ostium is calcified, upper limb access is generally preferred and nearly always performed on the left. The procedure can be started with a short, small-diameter sheath, such as a 10 cm 5-Fr or 6-Fr sheath, and a diagnostic catheter, allowing initial aortography. The sheath is then exchanged over the wire for a longer, wider sheath after assessment of the target vessel (typically 45–65 cm for the femoral approach, 90 cm for the brachial approach, and 100–110 cm for the radial approach) [44, 45]. An alternative is direct insertion of a 6-Fr to 8-Fr sheath that is long enough to reach the target vessel. When using these long sheaths, it is crucial to maintain their patency by either continuous infusion via the side arm or frequent aspiration and flushing to prevent in-sheath thrombosis.

To identify the origins of the CA and SMA, a flush catheter can be positioned in the abdominal aorta, just above the CA origin, to obtain antero-posterior and lateral angiograms [46]. Depending on mesenteric artery anatomy and access site (femoral or brachial/radial), various selective catheters and guidewires can be used to catheterise the target artery including a reverse directional catheter (e.g., Simmon’s or Cobra catheters) for the femoral route and an angled catheter (e.g., multipurpose catheter) for the radial/brachial route. Selective SMA/CA angiograms are performed in postero-anterior and lateral or oblique projections to confirm and analyse the arterial lesion.

For embolic AMI, aspiration can be performed manually, by applying a negative pressure using a large syringe attached to the catheter, or mechanically by using a powered aspiration pump system that delivers continuous and controlled suction; however, a recent research letter based on a small patient cohort reported no clear benefit of mechanical over manual aspiration in such a setting [47]. For manual aspiration, a sheath or a guiding catheter (e.g., 8-Fr with femoral access or 6-Fr with brachial and radial access) is usually first advanced through the sheath to position the tip at the origin of the SMA. A hydrophilic 0.035″ guidewire, or at minimum an 0.018″ guidewire, and an appropriate catheter is advanced through the embolus and introduced into a branch of the SMA, usually the ileocolic branch if the main trunk is occluded. To improve stability, a stiffer wire is substituted for the hydrophilic wire. A 5-Fr or 6-Fr guiding catheter is advanced co-axially through the 8-Fr guiding catheter beyond the occlusion. The guidewire is then withdrawn and aspiration is applied through the catheter using a 20- to 50-mL syringe while gradually withdrawing the catheter [18, 44]. This process can be repeated several times if necessary, until the embolus is completely removed or resists further aspiration.

Several studies, primarily small case series, have reported the use of mechanical thrombectomy systems in the treatment of embolic mesenteric ischaemia, with outcomes suggesting promising efficacy. Techniques adapted from neurointerventional stroke management—such as stent retriever deployment combined with manual aspiration and the use of dedicated suction catheters—have also been described [47, 48]. In addition, rheolytic and rotational thrombectomy devices have been utilised in limited patient cohorts [49, 50].

Additional procedures may be required. In patients with residual thromboembolic material and/or inadequate blood flow or when a thromboembolus is lodged in a distal vessel segment, catheter-directed thrombolysis may be performed. In addition, in the event of uncovering a residual luminal stenosis due to underlying atherosclerosis, a stent may be placed.

Catheter-directed thrombolysis involves placing an infusion catheter within or just proximal to the embolus. A multiple side-hole catheter, an end-hole catheter, or even a microcatheter can be used depending on whether the main trunk or a branch is involved. Local thrombolysis is then achieved by administering either recombinant tissue plasminogen activator at a rate of 0.5–1 mg/h or urokinase in various dosages (e.g., 120,000 IU/h) [18] depending on local availability. The patency of the target artery is then checked by serial angiograms once or twice a day [18]. Slow injection over 10–20 min of a urokinase bolus (e.g., 200,000–400,000 IU) has also been reported [44].

For atherosclerotic AMI or acute-on-chronic mesenteric ischaemia with occlusion of both the SMA and the CA, SMA recanalisation is the primary goal. If SMA recanalisation is not feasible or fails, the CA is targeted. The stenotic or occluded arterial segment is crossed using a hydrophilic 0.035″ or 0.018″ guidewire and an appropriate catheter. The intraluminal position of the catheter distal to the stenosis must be confirmed via angiography [51]. In the event of failure with the 0.035″ guidewire, a microguidewire (usually 0.014″) and microcatheter (2.0-Fr to 2.4-Fr) combination may be useful [51]. A stiffer guidewire is usually substituted for the hydrophilic guidewire to improve stability. Pre-dilatation of the stenosis can be performed using an undersized percutaneous transluminal angioplasty (PTA) balloon catheter, usually 3–4 mm in diameter, especially in patients with heavily calcified, hard plaques [46, 51]. An appropriately sized balloon-expandable stent, bare-metal stent, or covered stent is then deployed to cover the ostium and the lesion. The proximal stent may be extended with a second stent, usually a self-expanding nitinol stent, to cover long lesions [46]. For residual stenosis (> 30%), repeat PTA can be considered [46]. An angiogram is then performed to confirm adequate patency of the target vessel and peripheral branches with no residual stenosis > 30% [14, 51]. In the absence of strong evidence, some authors consider a trans-stent pressure gradient > 10 mmHg as a threshold for further angioplasty or stenting [52].

Following endovascular revascularisation, a planned second-look surgery is often advocated to assess bowel viability and determine the need for resection. In this context, diagnostic laparoscopy may be performed to evaluate bowel ischaemia and identify areas of irreversible damage, allowing for a tailored surgical resection [3, 18, 53, 54]. However, in the presence of initial or new clinical or radiological evidence of peritonitis or bowel infarction, immediate laparotomy is required [3].

### Chronic Mesenteric Ischaemia

Percutaneous stent placement is the current standard approach due to the higher technical success rate compared to PTA alone [10]. The SMA is the primary target. The CA is generally targeted when SMA recanalisation fails or is highly likely to fail due to heavy calcification, long lesions, or occlusion [18]. CA angioplasty or stenting should not be performed in patients with active compression by the median arcuate ligament [18].

The pressure gradient across the lesion can be assessed using a 4-Fr or 5-Fr catheter, either routinely or only when the severity of the stenosis is unclear [18, 52, 55, 56]. In general, severe SMA stenosis is defined as a mean arterial pressure gradient of 10 mmHg or more [18]. Others have considered a systolic pressure gradient of 20 mmHg or more to indicate significant stenosis (≥ 70%) [55].

After initial aortography and target vessel assessment, a long flexible 6-Fr or 7-Fr sheath is placed in or near the target vessel ostium. The CA or SMA is then catheterised using an appropriate catheter with a flexible hydrophilic guidewire [45]. Catheter position downstream of the stenosis must be confirmed by angiography [51]. An exchange catheter is placed and used to exchange the guidewire for a stiffer wire. Some authors prefer small-profile, stiff, 0.014″ or 0.018″ wires [57]. The long sheath is advanced over its dilator into the target artery. When advancement is not possible, a balloon 5 mm to 6 mm in diameter and 2 cm in length can be brought through the orifice of the artery and inflated. The sheath is then advanced into the artery as the balloon is deflated [45]. Systemic heparin must be administered before PTA or stenting. If necessary, the stenosis is pre-dilated (5–6 mm) [45, 46, 55]. Both monorail and over-the-wire PTA balloon systems can be used at this stage, depending on operator preference and lesion complexity. Monorail systems allow easier handling, while over-the-wire systems offer greater support, particularly in tortuous or calcified anatomy. A balloon-expandable stent is then positioned. A covered stent can be used for proximal SMA lesions if no side branches will be covered. Recent data support the use of covered stents rather than bare-metal stents, as they offer better primary patency [43]. The size and length of the stent are determined using angiography to measure the arterial diameter immediately distal to the target lesion and the length of the stenosis to be covered, allowing for proximal and distal extension beyond the lesion [46, 51]. The CA and SMA generally require stents 2–3 cm in length with a diameter of 5–7 mm and 6–8 mm, respectively. The stent must cover the ostium [46]. Approximately 5 mm of stent is allowed to protrude into the aorta. This intra-aortic segment is usually flared using a balloon 2 mm larger than the index balloon for the stent across the orifice [45]. While extending the stent into the aorta is generally required, it is preferable to avoid excessive protrusion when using a covered stent. An angiogram is then performed to check that the target vessel and peripheral branches are patent with no residual stenosis exceeding 30%. Repeated dilation may be considered for residual stenosis > 30%. The use of embolic protection devices to avoid distal embolisation when treating the SMA, particularly in patients with heavily calcified stenosis, occlusion, or an acute or subacute presentation, has been described but is not routinely used [55].

Although the IMA is considered the least important mesenteric artery, when both the SMA and CA are occluded and one of these arteries cannot be recanalised, stenting of the IMA may be considered to improve the collateral circulation [58, 59].

## Post-Treatment and Follow-Up Care

In both acute and chronic settings, immediate post-interventional care chiefly involves access site monitoring for evidence of haematoma or pseudo-aneurysm by physical examination and Duplex US as required. Haematoma-related nerve damage after brachial access must be detected early to avoid permanent sensory loss; thus, a focused physical examination for evidence of neural injury is essential.

### Acute Mesenteric Ischaemia

Even when endovascular treatment is technically successful, bowel resection may be required. Consequently, a planned second-look surgery following revascularisation is often performed to assess bowel viability [3, 18, 53, 54]. In contrast, when signs of peritonitis or bowel necrosis are evident clinically or radiologically, prompt surgical intervention with laparotomy and resection is required [3].

Although laboratory tests are non-specific, elevation of the L-lactate level, leucocyte count, and D-dimer level support the presence of ischaemia or necrosis when combined with suggestive symptoms and imaging findings [3]. The primary goal of intensive care for patients with AMI, irrespective of whether bowel resection has been performed, is to decompress the (remaining) bowel segments, improve their perfusion, and prevent the development of multi-organ failure [3]. Patients with systemic hypotension may require catecholamine (noradrenaline and dobutamine) therapy [3]. Damage to the mucosal barrier of the bowel carries a risk of bacterial translocation that warrants broad-spectrum antibiotic therapy, which should be continued after endovascular or open surgical intervention [3, 4].

Following invasive procedures, antiplatelet and/or anticoagulant therapy should be initiated as appropriate for the type of intervention used and any comorbidities (e.g., atrial fibrillation). Dual antiplatelet therapy for at least 1 month should be followed by lifelong monotherapy [3, 60]. In patients who do not have another condition requiring lifelong use, anticoagulant therapy should be given for 6 months [3, 4]. In a recent study, immediate post-operative parenteral anticoagulation improved outcomes after bowel resection in patients with AMI [61].

Duplex US monitoring of mesenteric artery patency 1, 6, and 12 months after the treatment and annually thereafter is recommended [3, 60]. Contrast-enhanced imaging is only indicated in cases of suspected recurrent bowel ischaemia [60].

Resection of a substantial length of small bowel can lead to short bowel syndrome and predicts prolonged parenteral nutrition and poor long-term survival [62]. In severe cases, intestinal transplantation may be an option [3, 62].

### Chronic Mesenteric Ischaemia

Post-interventional antiplatelet therapy is essential to prevent early stent thrombosis. Although no comparative studies have been conducted to identify the best pharmacotherapy regimen after angioplasty and/or stenting of visceral arteries, most interventionalists use the same medications as for renal or carotid artery stenting, namely, antiplatelet therapy with aspirin 100 mg daily lifelong combined during the 1st month with clopidogrel 75 mg daily [63].

Follow-up after angioplasty or stenting of a visceral artery consists mainly in clinical monitoring of changes in CMI symptoms such as post-prandial abdominal pain, weight loss, and intermittent diarrhoea. Follow-up imaging of the treated visceral artery can be performed at pre-defined intervals or only if typical symptoms recur. Duplex US and/or CTA may be performed depending on local practice and the patient’s general condition including body habitus and renal function. Magnetic resonance angiography is not indicated after visceral artery stenting given the major artifacts produced by stainless steel and cobalt-chrome stents. Prior to repeat angioplasty or stenting, recurrent stenosis should be confirmed with catheter angiography.

## Outcomes

### Acute Mesenteric Ischaemia

#### Procedural Outcomes

Compared with non-invasive treatment, endovascular interventions provide better overall results in patients with AMI. For example, in one study, the 30-day mortality rate was 81% with non-invasive treatment and 32% with endovascular therapy [64]. A review of studies published in 2013–2023 found that catheter-directed thrombolysis and/or thrombectomy (with or without stenting) had a technical success rate of 38.5–100%, a complication rate of 0–17%, an exploratory laparotomy rate of 9–73%, and a 30-day mortality rate of 15.3–53.8% [4]. Stenting had a technical success rate of 95–100%, an exploratory laparotomy rate of 36.8%, and a 30-day mortality rate of 42–62.5% [4].

### Procedure-Related Complications

Compared with open surgical revascularisation, endovascular therapy was associated with lower rates of wound infection, bowel necrosis, renal failure, respiratory failure and infection, myocardial infarction, multi-organ failure, and 30-day mortality in patients with AMI. In addition, patients with AMI who received endovascular therapy had shorter hospital stays and less often required total parenteral nutrition [3, 4, 65].

Independent predictors of post-procedural mortality in patients with AMI include age older than 70 years; comorbidities; prolonged symptom duration; hypotension; tachypnoea; hypoxia; failure of more than two organs; aetiology; elevation of the leukocyte count, serum lactate, bilirubin, and/or creatinine; involvement of more than one mesenteric artery; presence of porto-mesenteric vein gas; requirement for intra-operative fresh-frozen plasma transfusion; bowel resection longer than three feet; viable bowel remnant < 100 cm; need for second-look surgery; and surgical complications [66, 67].

### Long-Term Outcomes

In one study, the 1-year survival rate was 19% with non-invasive treatment and 52% with endovascular therapy [64]. The previous cited review of studies published in 2013–2023 found that catheter-directed thrombolysis and/or thrombectomy (with or without stenting) had a survival rate at 3 months, 1 year, and 3 years of 72.5–79%, 52–78%, and 54–63%, respectively [4]. Primary patency was 77–83% at 1 year, 69–76% at 2 years, and 45% at 5 years [4].

In two studies, no correlation was shown between the type of endovascular procedure performed and short- or long-term outcomes [9, 68]. When sub-groups of stents were analysed, covered stents were found to provide better patency than bare-metal stents [68, 69].

Few studies have evaluated the efficacy of hybrid approaches. The results suggest better outcomes than with either open surgical revascularisation or endovascular therapy alone [4, 65].

### Chronic Mesenteric Ischaemia

#### Procedural Outcomes

Recent data on procedural outcomes in high-volume centres show that visceral artery angioplasty and stenting have very high technical success rates of 90% to 100% [45, 46, 55, 63]. The main reasons for recanalisation failure are total calcified occlusion and long critical stenosis.

### Procedure-Related Complications

The main complications of visceral artery angioplasty or stenting are related to the access site, the most common being puncture-related haematoma and pseudo-aneurysm. After brachial artery access, nerve damage with sensory or motor loss is less common but serious. Stent dislodgement or dislocation is rare [43]; pull-back over the guidewire and deployment in the iliac artery may be a valuable treatment option in this situation. Lastly, catheter- or wire-related vessel-wall dissection potentially resulting in acute thrombosis of the injured vessel is a serious but very rare procedure-related complication [43].

### Long-Term Outcomes

The in situ (in-stent) restenosis rate after balloon angioplasty and stent placement in visceral arteries is relatively high, ranging between 30% and 45%. Restenosis and reocclusion are more common after CA compared to SMA stenting. In a large retrospective cohort study, the 1-year primary patency rate was 18% and 55% for the CA and SMA, respectively [70]. In addition, progression of atherosclerotic disease distal to the index stenosis may result in clinical recurrence. A case report suggests that drug-eluting stents may help to prevent recurrence [71]; however, there are no studies comparing drug-eluting to bare-metal stents for mesenteric occlusive disease. In a retrospective study of covered stents to restore SMA patency, the primary patency, primary-assisted patency, and secondary patency rates at 2 years were 76%, 95%, and 95%, respectively [69]. In addition, several comparative studies demonstrated better patency rates with covered versus bare-metal stents for chronic mesenteric atherosclerosis. Patency rates at 3 years were 92 ± 6% with covered stents and 52 ± 5% with bare-metal stents in a retrospective cohort [72]. A multicentre randomised trial comparing bare-metal to covered stents was published in 2024 [43]. Two years after SMA stenting, the patency rate was 81% with covered stents and 49% with bare-metal stents (*p* < 0.0001). Lastly, in the rare case of chronic SMA and/or CA occlusion with severe ostial or post-ostial stenosis of the IMA, angioplasty and stenting of the latter may be effective [43, 59].

## Concluding Statement

AMI is a life-threatening condition that requires rapid diagnostic and multidisciplinary management. First-line endovascular revascularisation is indicated for haemodynamically stable patients with no evidence of transmural necrosis or peritonitis. The choice of recanalisation technique depends chiefly on the cause of arterial occlusion: stenting is used for atherosclerosis and thrombus aspiration for emboli, with additional catheter-directed thrombolysis or stenting if necessary. In the presence of intestinal necrosis and peritonitis, bowel resection is necessary, failing which, revascularisation remains necessary to improve the chances of salvaging ischaemic but non-necrotic bowel segments. The relative timing of revascularisation and bowel resection is still a matter of debate.

CMI requires a multidisciplinary approach to secure the diagnosis. Endovascular revascularisation with stenting is now the primary treatment method, with covered stents appearing to demonstrate better patency rates than bare-metal stents.

## Abbreviations

AMI: Acute mesenteric ischaemia
CA: Coeliac artery
CMI: Chronic mesenteric ischaemia
IMA: Inferior mesenteric artery
PTA: Percutaneous transluminal angioplasty
SMA: Superior mesenteric artery

## References

[1] Tamme K, Reintam Blaser A, Laisaar KT, Mändul M, Kals J, Forbes A, Kiss O, Acosta S, Bjørck M, Starkopf J. Incidence and outcomes of acute mesenteric ischaemia: a systematic review and meta-analysis. BMJ Open. 2022;12(10): e062846. 10.1136/bmjopen-2022-062846.PMID:36283747;PMCID:PMC9608543. 36283747 10.1136/bmjopen-2022-062846 PMC9608543

[2] Magnus L, Lejay A, Philouze G, Chakfé N, Collange O, Thaveau F, et al. Mortality and delays of management of acute mesenteric ischemia: the need of a dedicated program. Ann Vasc Surg. 2023;91:28–35. 36549474 10.1016/j.avsg.2022.12.070

[3] Bala M, Catena F, Kashuk J, De Simone B, Gomes CA, Weber D, et al. Acute mesenteric ischemia: updated guidelines of the World Society of Emergency Surgery. World J Emerg Surg WJES. 2022;17:54. 36261857 10.1186/s13017-022-00443-x PMC9580452

[4] Gries JJ, Virk HUH, Chen B, Sakamoto T, Alam M, Krittanawong C. Advancements in revascularization strategies for acute mesenteric ischemia: a comprehensive review. J Clin Med. 2024;13:570. 38276076 10.3390/jcm13020570 PMC10816895

[5] El Farargy M, Abdel Hadi A, Abou Eisha M, Bashaeb K, Antoniou GA. Systematic review and meta-analysis of endovascular treatment for acute mesenteric ischaemia. Vascular. 2017;25:430–8. 28121281 10.1177/1708538116689353

[6] Tilsed JV, Casamassima A, Kurihara H, Mariani D, Martinez I, Pereira J, et al. ESTES guidelines: acute mesenteric ischaemia. Eur J Trauma Emerg Surg. 2016;42:253–70. 26820988 10.1007/s00068-016-0634-0 PMC4830881

[7] Sermonesi G, Tian BWCA, Vallicelli C, Abu-Zidan FM, Damaskos D, Kelly MD, et al. Cesena guidelines: WSES consensus statement on laparoscopic-first approach to general surgery emergencies and abdominal trauma. World J Emerg Surg. 2023;18:57. 38066631 10.1186/s13017-023-00520-9 PMC10704840

[8] Xhepa G, Vanzulli A, Sciacqua LV, Inzerillo A, Faerber P, Ierardi AM, et al. Advancements in treatment strategies for chronic mesenteric ischemia: a comprehensive review. J Clin Med. 2023;12:7112. 38002726 10.3390/jcm12227112 PMC10672107

[9] Kolkman JJ, Geelkerken RH. Diagnosis and treatment of chronic mesenteric ischemia: an update. Best Pract Res Clin Gastroenterol. 2017;31:49–57. 28395788 10.1016/j.bpg.2017.01.003

[10] Terlouw LG, Moelker A, Abrahamsen J, Acosta S, Bakker OJ, Baumgartner I, et al. European guidelines on chronic mesenteric ischaemia - joint United European gastroenterology, European association for gastroenterology, endoscopy and nutrition, european society of gastrointestinal and abdominal radiology, netherlands association of hepatogastroenterologists, Hellenic society of gastroenterology, cardiovascular and interventional radiological society of Europe, and Dutch mesenteric ischemia study group clinical guidelines on the diagnosis and treatment of patients with chronic mesenteric ischaemia. United Eur Gastroenterol J. 2020;8:371–95. 10.1177/2050640620916681 PMC7226699 32297566

[11] Anandan AS, Silva M. Chronic mesenteric ischemia: diagnosis & management. Ann Med Surg. 2022;80: 104138. 10.1016/j.amsu.2022.104138 PMC9422046 36045857

[12] Wilkins LR, Stone JR. Chronic mesenteric ischemia. Tech Vasc Interv Radiol. 2015;18:31–7. 25814201 10.1053/j.tvir.2014.12.005

[13] Huber TS, Björck M, Chandra A, Clouse WD, Dalsing MC, Oderich GS, et al. Chronic mesenteric ischemia: clinical practice guidelines from the society for vascular surgery. J Vasc Surg. 2021;73:87S-115S. 33171195 10.1016/j.jvs.2020.10.029

[14] Pillai AK, Kalva SP, Hsu SL, Walker TG, Silberzweig JE, Annamalai G, et al. Quality improvement guidelines for mesenteric angioplasty and stent placement for the treatment of chronic mesenteric ischemia. J Vasc Interv Radiol. 2018;29:642–7. 29574024 10.1016/j.jvir.2017.11.024

[15] Omran S, Konietschke F, Mueller V, de Bucourt M, Frese JP, Greiner A. Development of a novel scoring model to estimate the severity grade of mesenteric artery stenosis. J Clin Med. 2022;11:7420. 36556035 10.3390/jcm11247420 PMC9785168

[16] Pecoraro F, Rancic Z, Lachat M, Mayer D, Amann-Vesti B, Pfammatter T, et al. Chronic mesenteric ischemia: critical review and guidelines for management. Ann Vasc Surg. 2013;27:113–22. 23088809 10.1016/j.avsg.2012.05.012

[17] Gupta PK, Horan SM, Turaga KK, Miller WJ, Pipinos II. Chronic mesenteric ischemia: endovascular versus open revascularization. J Endovasc Ther. 2010;17:540–9. 20681773 10.1583/09-2935.1

[18] Björck M, Koelemay M, Acosta S, Bastos Goncalves F, Kölbel T, Kolkman JJ, et al. Editor’s choice—management of the diseases of mesenteric arteries and veins: clinical practice guidelines of the European society of vascular surgery (ESVS). Eur J Vasc Endovasc Surg. 2017;53:460–510. 28359440 10.1016/j.ejvs.2017.01.010

[19] Nuzzo A, Peoc’h K, Vaittinada Ayar P, Tran-Dinh A, Weiss E, Panis Y, et al. Improving clinical suspicion of acute mesenteric ischemia among patients with acute abdomen: a cross-sectional study from an intestinal stroke center. World J Emerg Surg. 2023;18:37. 37287011 10.1186/s13017-023-00505-8 PMC10246417

[20] Copin P, Zins M, Nuzzo A, Purcell Y, Beranger-Gibert S, Maggiori L, et al. Acute mesenteric ischemia: a critical role for the radiologist. Diagn Interv Imaging. 2018;99:123–34. 29433829 10.1016/j.diii.2018.01.004

[21] Garzelli L, Nuzzo A, Copin P, Calame P, Corcos O, Vilgrain V, et al. Contrast-enhanced CT for the diagnosis of acute mesenteric ischemia. AJR Am J Roentgenol. 2020;215:29–38. 32374661 10.2214/AJR.19.22625

[22] Kärkkäinen JM. Acute mesenteric ischemia in elderly patients. Expert Rev Gastroenterol Hepatol. 2016;10:985–8. 27411031 10.1080/17474124.2016.1212657

[23] Carver TW, Vora RS, Taneja A. Mesenteric ischemia. Crit Care Clin. 2016;32:155–71. 27016159 10.1016/j.ccc.2015.11.001

[24] Kärkkäinen JM. Acute mesenteric ischemia: a challenge for the acute care surgeon. Scand J Surg. 2021;110:150–8. 33866891 10.1177/14574969211007590 PMC8258713

[25] Cudnik MT, Darbha S, Jones J, Macedo J, Stockton SW, Hiestand BC. The diagnosis of acute mesenteric ischemia: a systematic review and meta-analysis. Acad Emerg Med. 2013;20:1087–100. 24238311 10.1111/acem.12254

[26] Sgourakis G, Papapanagiotou A, Kontovounisios C, Karamouzis MV, Lanitis S, Konstantinou C, et al. The value of plasma neurotensin and cytokine measurement for the detection of bowel ischaemia in clinically doubtful cases: a prospective study. Exp Biol Med (Maywood). 2013;238:874–80. 23828592 10.1177/1535370213494663

[27] Ludewig S, Jarbouh R, Ardelt M, Mothes H, Rauchfuß F, Fahrner R, et al. Bowel ischemia in ICU patients: diagnostic value of I-FABP depends on the interval to the triggering event. Gastroenterol Res Pract. 2017;2017:2795176. 28630622 10.1155/2017/2795176 PMC5467337

[28] Reintam Blaser A, Starkopf J, Björck M, Forbes A, Kase K, Kiisk E, et al. Diagnostic accuracy of biomarkers to detect acute mesenteric ischaemia in adult patients: a systematic review and meta-analysis. World J Emerg Surg. 2023;18:44. 37658356 10.1186/s13017-023-00512-9 PMC10474684

[29] Expert Panels on Vascular Imaging and Gastrointestinal Imaging: Ginsburg M, Obara P, Lambert DL, Hanley M, Steigner ML, et al. ACR appropriateness criteria^®^ imaging of mesenteric ischemia. J Am Coll Radiol. 2018;15:S332–40 10.1016/j.jacr.2018.09.018 30392602

[30] Kanasaki S, Furukawa A, Fumoto K, Hamanaka Y, Ota S, Hirose T, et al. Acute mesenteric ischemia: multidetector CT findings and endovascular management. Radiographics. 2018;38:945–61. 29757725 10.1148/rg.2018170163

[31] Lourenco PDM, Rawski R, Mohammed MF, Khosa F, Nicolaou S, McLaughlin P. Dual-energy CT iodine mapping and 40-keV monoenergetic applications in the diagnosis of acute bowel ischemia. AJR Am J Roentgenol. 2018;211:564–70. 29927328 10.2214/AJR.18.19554

[32] ter Steege RWF, Sloterdijk HS, Geelkerken RH, Huisman AB, van der Palen J, Kolkman JJ. Splanchnic artery stenosis and abdominal complaints: clinical history is of limited value in detection of gastrointestinal ischemia. World J Surg. 2012;36:793–9. 22354487 10.1007/s00268-012-1485-4 PMC3299959

[33] Björnsson S, Resch T, Acosta S. Symptomatic mesenteric atherosclerotic disease-lessons learned from the diagnostic workup. J Gastrointest Surg. 2013;17:973–80. 23307340 10.1007/s11605-013-2139-z

[34] Oliva IB, Davarpanah AH, Rybicki FJ, Desjardins B, Flamm SD, Francois CJ, et al. ACR appropriateness criteria® imaging of mesenteric ischemia. Abdom Imaging. 2013;38:714–9. 23296712 10.1007/s00261-012-9975-2

[35] Corcos O, Castier Y, Sibert A, Gaujoux S, Ronot M, Joly F, et al. Effects of a multimodal management strategy for acute mesenteric ischemia on survival and intestinal failure. Clin Gastroenterol Hepatol. 2013;11:158-165.e2. 23103820 10.1016/j.cgh.2012.10.027

[36] Molyneux K, Beck-Esmay J, Koyfman A, Long B. High risk and low prevalence diseases: mesenteric ischemia. Am J Emerg Med. 2023;65:154–61. 36638612 10.1016/j.ajem.2023.01.001

[37] Bala M, Kashuk J, Moore EE, Kluger Y, Biffl W, Gomes CA, et al. Acute mesenteric ischemia: guidelines of the world society of emergency surgery. World J Emerg Surg. 2017;12:38. 28794797 10.1186/s13017-017-0150-5 PMC5545843

[38] Wyers MC. Acute mesenteric ischemia: diagnostic approach and surgical treatment. Semin Vasc Surg. 2010;23:9–20. 20298945 10.1053/j.semvascsurg.2009.12.002

[39] Corcos O, Nuzzo A. Gastro-intestinal vascular emergencies. Best Pract Res Clin Gastroenterol. 2013;27:709–25. 24160929 10.1016/j.bpg.2013.08.006

[40] Silvestri L, van Saene HKF, Zandstra DF, Marshall JC, Gregori D, Gullo A. Impact of selective decontamination of the digestive tract on multiple organ dysfunction syndrome: systematic review of randomized controlled trials. Crit Care Med. 2010;38:1370–6. 20308882 10.1097/CCM.0b013e3181d9db8c

[41] Memarian S, Krokidis M, O’Sullivan G, Peynircioglu B, Rossi M, Kashef E. CIRSE standards of practice on arterial access for interventions. Cardiovasc Intervent Radiol. 2023;46:302–9. 36705706 10.1007/s00270-022-03349-y

[42] van Dijk LJD, van Noord D, van Mierlo M, Bijdevaate DC, Bruno MJ, Moelker A. Single-center retrospective comparative analysis of transradial, transbrachial, and transfemoral approach for mesenteric arterial procedures. J Vasc Interv Radiol. 2020;31:130–8. 31771892 10.1016/j.jvir.2019.08.026

[43] Terlouw LG, van Dijk LJD, van Noord D, Bakker OJ, Bijdevaate DC, Erler NS, et al. Covered versus bare-metal stenting of the mesenteric arteries in patients with chronic mesenteric ischaemia (CoBaGI): a multicentre, patient-blinded and investigator-blinded, randomised controlled trial. Lancet Gastroenterol Hepatol. 2024;9:299–309. 38301673 10.1016/S2468-1253(23)00402-8

[44] Jia Z, Jiang G, Tian F, Zhao J, Li S, Wang K, et al. Early endovascular treatment of superior mesenteric occlusion secondary to thromboemboli. Eur J Vasc Endovasc Surg. 2014;47:196–203. 24183620 10.1016/j.ejvs.2013.09.025

[45] Haben C, Park WM, Bena JF, Parodi FE, Lyden SP. Improving midterm results justify the continued use of bare-metal stents for endovascular therapy for chronic mesenteric ischemia. J Vasc Surg. 2020;71:111–20. 31327617 10.1016/j.jvs.2019.01.094

[46] Altintas Ü, Lawaetz M, de la Motte L, Riazi H, Lönn L, Lindh M, et al. Endovascular treatment of chronic and acute on chronic mesenteric ischaemia: results from a national cohort of 245 cases. Eur J Vasc Endovasc Surg. 2021;61:603–11. 33589326 10.1016/j.ejvs.2021.01.003

[47] Garzelli L, Ben Abdallah I, Nuzzo A, Corcos O, Castier Y, Ronot M. Endovascular thrombectomy for acute arterial mesenteric ischaemia: no benefit of mechanical over manual thrombus aspiration. Eur J Vasc Endovasc Surg. 2022;64:128–9. 35568314 10.1016/j.ejvs.2022.05.020

[48] Shi Y, Gu J, Chen L, Shi W, Ahmed MJ, Huang H, Su H. Mechanical thrombectomy using the solitaire AB device for acute embolic mesenteric ischemia. J Vasc Interv Radiol. 2019;30:43–8. 30527655 10.1016/j.jvir.2018.08.005

[49] Zhang Z, Chen X, Li C, Feng H, Yu H, Zhu R. Percutaneous mechanical thrombectomy for acute superior mesenteric artery embolism: preliminary experience in five cases. Ann Vasc Surg. 2020;63:186–92. 31629130 10.1016/j.avsg.2019.08.096

[50] Ballehaninna UK, Hingorani A, Ascher E, Shiferson A, Marks N, Aboian E, et al. Acute superior mesenteric artery embolism: reperfusion with AngioJet hydrodynamic suction thrombectomy and pharmacologic thrombolysis with the EKOS catheter. Vascular. 2012;20:166–9. 22442382 10.1258/vasc.2011.cr0311

[51] Pedersoli F, Schönau K, Schulze-Hagen M, Keil S, Isfort P, Gombert A, et al. Endovascular revascularization with stent implantation in patients with acute mesenteric ischemia due to acute arterial thrombosis: clinical outcome and predictive factors. Cardiovasc Intervent Radiol. 2021;44:1030–8. 33825061 10.1007/s00270-021-02824-2 PMC8190006

[52] Dias NV, Acosta S, Resch T, Sonesson B, Alhadad A, Malina M, et al. Mid-term outcome of endovascular revascularization for chronic mesenteric ischaemia. Br J Surg. 2010;97:195–201. 20035543 10.1002/bjs.6819

[53] Sakamoto T, Kubota T, Funakoshi H, Lefor AK. Multidisciplinary management of acute mesenteric ischemia: surgery and endovascular intervention. World J Gastrointest Surg. 2021;13:806–13. 34512904 10.4240/wjgs.v13.i8.806 PMC8394382

[54] Estler A, Estler E, Feng YS, Seith F, Wießmeier M, Archid R, et al. Treatment of acute mesenteric ischemia: Individual challenges for interventional radiologists and abdominal surgeons. J Pers Med. 2022;13:55. 36675716 10.3390/jpm13010055 PMC9864352

[55] Aburahma AF, Campbell JE, Stone PA, Hass SM, Mousa AY, Srivastava M, et al. Perioperative and late clinical outcomes of percutaneous transluminal stentings of the celiac and superior mesenteric arteries over the past decade. J Vasc Surg. 2013;57:1052–61. 23332984 10.1016/j.jvs.2012.10.082

[56] Landis MS, Rajan DK, Simons ME, Hayeems EB, Kachura JR, Sniderman KW. Percutaneous management of chronic mesenteric ischemia: outcomes after intervention. J Vasc Interv Radiol. 2005;16:1319–25. 16221902 10.1097/01.RVI.0000171697.09811.0E

[57] Mendes BC, Oderich GS, Tallarita T, Kanamori KS, Kalra M, DeMartino RR, et al. Superior mesenteric artery stenting using embolic protection device for treatment of acute or chronic mesenteric ischemia. J Vasc Surg. 2018;68:1071–8. 29685508 10.1016/j.jvs.2017.12.076

[58] Wohlauer M, Kobeiter H, Desgranges P, Becquemin JP, Cochennec F. Inferior mesenteric artery stenting as a novel treatment for chronic mesenteric ischemia in patients with an occluded superior mesenteric artery and celiac trunk. Eur J Vasc Endovasc Surg. 2014;27:e21–3. 24920877 10.1016/j.ejvsextra.2014.01.001 PMC4049346

[59] Nair M, Kingsmore D, Hennessy M, Hussey K. Stenting the inferior mesenteric artery for chronic mesenteric ischaemia. Eur J Vasc Endovasc Surg. 2023;65:159–60. 36216235 10.1016/j.ejvs.2022.10.006

[60] Scallan OH, Duncan AA. Current approaches for mesenteric ischemia and visceral aneurysms. Surg Clin North Am. 2023;103:703–31. 37455033 10.1016/j.suc.2023.04.017

[61] Liu H-T, Lai C-Y, Liao J-J, Chen Y-J, Cheng S-B, Wu C-C. Immediate postoperative parenteral anticoagulant therapy in patients with mesenteric ischemia after intestinal resection: a retrospective cohort study at a single institute. BMC Gastroenterol. 2023;23:56. 36890480 10.1186/s12876-023-02691-w PMC9996985

[62] Lemma A, Pikkarainen S, Pohju A, Tolonen M, Mentula P, Vikatmaa P, et al. Potential for intestinal transplantation after acute mesenteric ischemia in patients aged less than 70 years: a population-based study. Scand J Surg. 2023;112:77–85. 36755514 10.1177/14574969231151374

[63] Awouters J, Jardinet T, Hiele M, Laenen A, Dymarkowski S, Fourneau I, et al. Factors predicting long-term outcomes of percutaneous angioplasty and stenting of the superior mesenteric artery for chronic mesenteric ischemia. Vasa. 2021;50:431–8. 34231372 10.1024/0301-1526/a000964

[64] Pengermä P, Venesmaa S, Karjalainen J, Ukkonen M, Saari P, Kärkkäinen JM. Long-term outcome after implementation of endovascular-first strategy to treat acute mesenteric ischemia. J Vasc Surg. 2023;78:1524–30. 37586616 10.1016/j.jvs.2023.08.100

[65] Zhao Y, Yin H, Yao C, Deng J, Wang M, Li Z, et al. Management of acute mesenteric ischemia: a critical review and treatment algorithm. Vasc Endovascular Surg. 2016;50:183–92. 27036673 10.1177/1538574416639151

[66] Dhamnaskar SS, Sawarkar PC, Mandal S, Vijaykumaran P. Predictors of mortality in acute mesenteric vascular ischemia with bowel gangrene. Int Surg J. 2016;3:1996–2002.

[67] Otto CC, Czigany Z, Heise D, Bruners P, Kotelis D, Lang SA, et al. Prognostic factors for mortality in acute mesenteric ischemia. J Clin Med. 2022;11:3619. 35806904 10.3390/jcm11133619 PMC9267588

[68] Zhou Y, Ryer EJ, Garvin RP, Pham A, Irvan JL, Orlova K, et al. Outcomes of endovascular treatments for in-stent restenosis in patients with mesenteric atherosclerotic disease. J Vasc Surg. 2019;69:833–42. 30528413 10.1016/j.jvs.2018.08.166

[69] Girault A, Pellenc Q, Roussel A, Senemaud J, Cerceau P, Maggiori L, et al. Midterm results after covered stenting of the superior mesenteric artery. J Vasc Surg. 2021;74:902-909.e3. 33684478 10.1016/j.jvs.2021.02.038

[70] Ahanchi SS, Stout CL, Dahl TJ, Carty RL, Messerschmidt CA, Panneton JM. Comparative analysis of celiac versus mesenteric artery outcomes after angioplasty and stenting. J Vasc Surg. 2013;57:1062–6. 23313180 10.1016/j.jvs.2012.10.081

[71] Cardaioli P, Rigatelli G, Zattoni L, Giordan M. Drug-eluting stent for recurrent mesenteric artery in-stent restenosis. J Endovasc Ther. 2007;14:748–51. 17924744 10.1177/152660280701400522

[72] Oderich GS, Erdoes LS, Lesar C, Mendes BC, Gloviczki P, Cha S, et al. Comparison of covered stents versus bare metal stents for treatment of chronic atherosclerotic mesenteric arterial disease. J Vasc Surg. 2013;58:1316–23. 23827340 10.1016/j.jvs.2013.05.013

